# Nonlinear dynamics of diamagnetically levitating resonators

**DOI:** 10.1007/s11071-024-10018-x

**Published:** 2024-07-20

**Authors:** Xianfeng Chen, Tjebbe de Lint, Farbod Alijani, Peter G. Steeneken

**Affiliations:** 1 Department of Precision and Microsystems Engineering, Delft University of Technology, Mekelweg 2, 2628 CD Delft, The Netherlands; 2 A*STAR Quantum Innovation Centre (Q.InC), Institute for Materials Research and Engineering (IMRE), Agency for Science, Technology and Research (A*STAR), 2 Fusionopolis Way, 08-03, Innovis, 138634 Singapore, Singapore; 3 Kavli Institute of Nanoscience, Delft University of Technology, Lorentzweg 1, 2628 CJ Delft, The Netherlands

**Keywords:** Nonlinear dynamics, Diamagnetic levitation, Magnetic force, Nonlinear damping

## Abstract

The ultimate isolation offered by levitation provides new opportunities for studying fundamental science and realizing ultra-sensitive floating sensors. Among different levitation schemes, diamagnetic levitation is attractive because it allows stable levitation at room temperature without a continuous power supply. While the dynamics of diamagnetically levitating objects in the linear regime are well studied, their nonlinear dynamics have received little attention. Here, we experimentally and theoretically study the nonlinear dynamic response of graphite resonators that levitate in permanent magnetic traps. By large amplitude actuation, we drive the resonators into nonlinear regime and measure their motion using laser Doppler interferometry. Unlike other magnetic levitation systems, here we observe a resonance frequency reduction with amplitude in a diamagnetic levitation system that we attribute to the softening effect of the magnetic force. We then analyze the asymmetric magnetic potential and construct a model that captures the experimental nonlinear dynamic behavior over a wide range of excitation forces. We also investigate the linearity of the damping forces on the levitating resonator, and show that although eddy current damping remains linear over a large range, gas damping opens a route for tuning nonlinear damping forces via the squeeze-film effect.

**Supplementary Information:**

The online version contains supplementary material available at 10.1007/s11071-024-10018-x.

## Introduction

Gaining control over the dynamics of levitating objects has been a long-sought after goal, both because contactless levitation provides extreme isolation from external sources of heat and friction and because it allows six degrees-of-freedom rigid body motion. Recently, the interest in the field of levitodynamics [1] has surged, stimulated by the demonstration of quantum ground state cooling [2, 3] and the use of extremely high-Q levitating resonators for highly-sensitive sensors [4–6]. Among different levitation schemes, diamagnetic levitation has the advantage of being the only passive one that does not require continuous energy supply or cryogenic temperatures to realize levitation [5, 7–10], thus differentiating it from other kinds of levitation mechanisms including optical, superconducting and electrical levitation [1, 11]. Interestingly, the passive, zero power nature of diamagnetic levitation does not incur heating and noise that can be limits in optical and electrical schemes [4, 12, 13]. Moreover, suitably designed magnetic traps from permanent magnets allow stable diamagnetic levitation in high vacuum without active feedback [8], enabling levitation of high-mass macroscopic objects, that provide increased sensitivity in inertial sensors [14], accelerometers [15] and gravitational field sensors [16, 17].

The low-amplitude rigid body dynamics of diamagnetically levitating resonators in the linear regime is pretty well-known [5, 7, 18]. However, the linearity of the response in these devices cannot be sustained indefinitely due to the nonlinear nature of the magnetic field. Since damping forces are small in these levitating systems, small forces are often sufficient to drive them into the relatively uncharted nonlinear regime [19, 20]. Although several studies have already explored the nonlinear dynamics of magnetically levitating objects [21–23], these investigations primarily focused on systems where the levitation force arises from magnet-to-magnet interactions. In contrast, the nonlinear behavior of magnet-to-diamagnet interactions, that allow stable levitation without active feedback, has received comparatively little attention. Therefore, a good understanding of the nonlinear effects that govern the dynamics of diamagnetically levitating resonators in the high-amplitude regime is of importance, especially in applications like levitating mirrors, translation stages, and rotors [8, 24–27]. Moreover, this understanding can provide a route for using nonlinear dynamics to analyze levitation force fields.

Here, we study the nonlinear dynamics of diamagnetic graphite plates that stably levitate in a magnetic trap formed by four permanent magnets. By measuring the frequency response of the plates in vacuum and driving their motion by base excitation, the nonlinearity of the resonant motion is determined and analyzed. By characterizing the magnetic force that maintains the levitation we show that the magnetic potential is the largest source of nonlinearity. By fitting the experimental data we further highlight that the nonlinear dynamics of diamagnetically levitating objects deviates from the common Duffing oscillator response and is best described by a nonlinear stiffness function of quintic order. Finally, we discuss the sources of nonlinear damping in our measurements, and show that even though eddy current damping remains linear, squeeze-film effect contributes significantly to the observed dissipation. However, the model quickly deviates from experiments as the amplitude of oscillations increases, thus suggesting that the squeeze-film formulation for nonlinear dynamics of levitating objects shall be revisited.

## Results

### Experimental methods

**Fig. 1:**
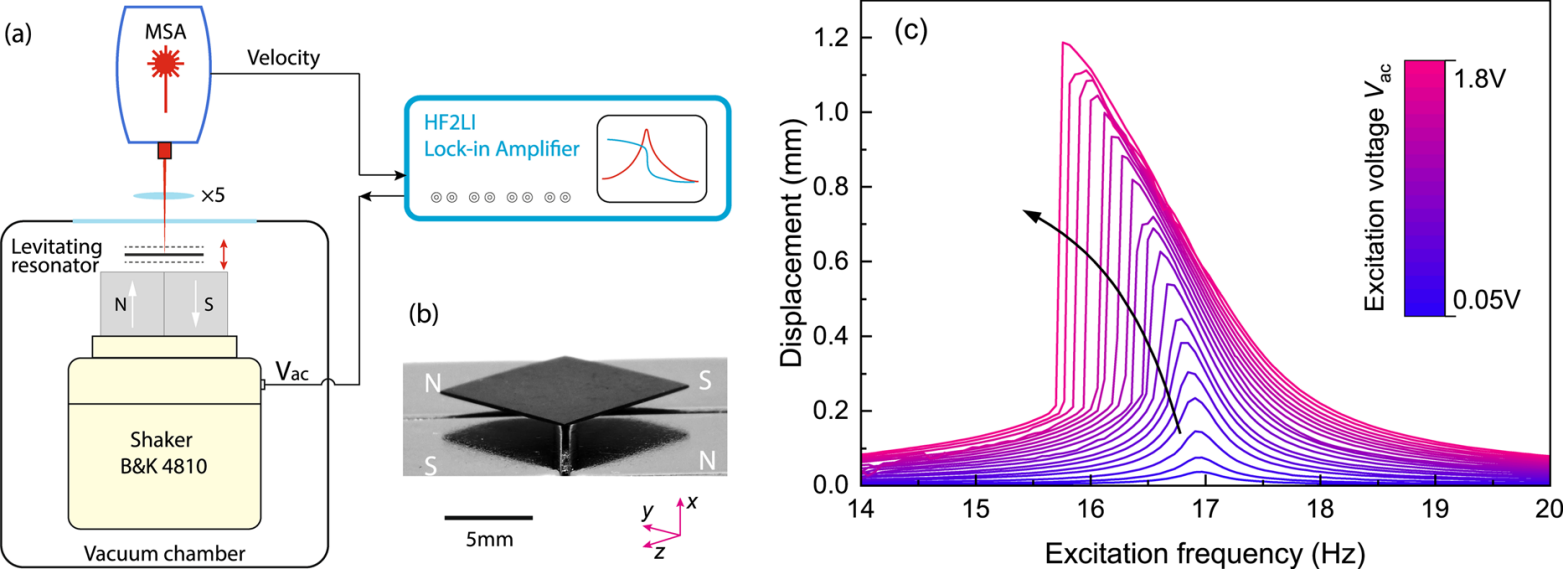
Measurement setup and nonlinear frequency response of a diamagnetically levitating plate. **a** Schematic of the experimental setup consisting of a Polytec Laser Doppler Vibrometer MSA400 for velocity readout and a Bruel and Kjaer shaker 4810 for base excitation. The excitation voltage is generated by a Zurich lock-in amplifier HF2LI that drives the levitating plate to vibrate in the vertical direction. The vibration signal is recorded by the MSA vibrometer and transferred to the lock-in amplifier for signal analysis. **b** Image of a $10\times 10\times {0.28}\,{\hbox {mm}^3}$ plate levitating over four 12 mm cubic NdFeB magnets with alternating magnetization, where N stands for north pole and S stands for south pole. Included is the defined coordinate system with *x* as the vertical direction. **c** Frequency response curves of the levitating plate excited by different driving voltage when backwards sweeping the frequency from 20 to 14 Hz

The magnetic levitation system used in our experiments consists of a pyrolytic graphite plate and four permanent magnets, as shown in Fig. 1b. The graphite is purchased from MTI Corporation and cut into a $10\times 10\times {0.28}\,{\hbox {mm}^{3}}$ plate using a Optec micro laser cutter, after which its surface is polished using a sand paper with 5 $\upmu$m grains to improve the surface quality for optical measurements. The plate levitates stably above four cubic NdFeB magnets in a checkerboard arrangement with alternating out-of-plane magnetization (Fig. 1b). In the minimum magnetic and gravitational potential, the plate edges have a 45$^\circ$ angle with the magnet edges naturally. The natural levitation gap where the gravitational force of the plate equals the magnetic force is $H_0={1.18}$ mm, as measured by a Keyence digital microscope (VHX-6000).

To drive the levitating plate into motion, we use a mini shaker (B &K 5810) which we attach under the magnets as shown in Fig. 1a. To detect the motion of the levitating plate, we then use a Polytec Laser Doppler Vibrometer and measure the out-of-plane velocity of the plate. The spectral response of the plate is obtained by sweeping the excitation frequency around the plate’s resonance using a Zurich Lock-in Amplifier. To eliminate effects [7] of air damping, we conduct our measurements in a vacuum chamber at a pressure below $10^{-4}$ mbar. Figure 1c shows the frequency response of the levitating plate when sweeping the excitation frequency downward from 20 to 14 Hz, for different driving voltages. When the driving voltage is small $V_{\textrm{ac}}={0.05}$ V, the frequency response of the plate is linear with a resonance frequency of $f_{\textrm{r}}={16.9}$ Hz and a quality factor $Q_{\textrm{L}}=48$ obtained by fitting the measured linear frequency responses of the levitating resonator with a Lorenzian function. With the excitation voltage increasing from 0.05 to 1.8 V, the peak frequency decreases and the displacement amplitude increases. Since the natural levitation gap is $H_0={1.18}$ mm, the plate almost touches the magnets when driving with 1.8 V at the peak frequency, obtaining a displacement amplitude close to $H_0$ (see Fig. 1c). In the following we keep the driving voltage on the shaker below 1.8 V, to prevent impact of the plate on the magnets. The observed dynamics of the diamagnetic plate is clearly nonlinear, resembling a Duffing resonator with negative nonlinear stiffness. To analyze the origin of this nonlinear dynamics, characterization of the magnetic forces is needed.

### Nonlinear magnetic force

**Fig. 2:**
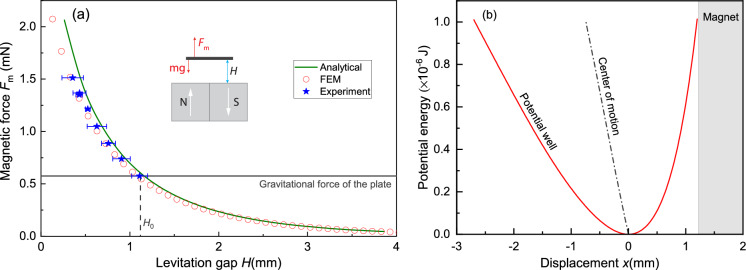
Position-dependent nonlinear magnetic force on the levitating plate. **a** The total mass $m_{\textrm{tot}}$ of the levitating pyrolytic graphite plate is varied by adding non-magnetic polymer blocks. For each datapoint the levitation gap *H* between the bottom of the plate and the top of the permanent magnets is measured using a Keyence digital microscope, while the magnetic force is determined from the static equilibrium with the gravitational force $|F_m|=m_{\textrm{tot}}g$. $H_0$ stands for the natural levitation gap of the plate without adding polymer blocks. Experimental data are compared to FEM and analytical simulations. **b** Potential energy of the plate as a function of its displacement *x* ($x=H_0-H$) based on the FEM $F_{\textrm{m}}-H$ curve in Fig. 2a. The dot-dash line shows its center of motion (midpoint between maximum and minimum displacement) when the plate is in free vibration

To analyze the nonlinear dynamic behaviour of the levitating plate, we first determine the stiffness of the magnetic force using experimental and analytic methods. As shown in the inset of Fig. 2a, only two forces are acting on the plate when it is levitating in static equilibrium: the magnetic force and the gravitational force. Since the gravitational force is independent of the levitating object’s displacement, only the magnetic force influences the plate’s stiffness.

To determine the position dependent magnetic force experimentally, we add non-magnetic polymer blocks with different weights on top of the graphite plate and measure the mass $m_{\textrm{tot}}$ of the plate with blocks, from which we determine the magnetic force in static equilibrium from $F_m=m_{\textrm{tot}} g$. For each mass value, we measure the levitation gap *H* between the plate and magnet using a Keyence digital microscope. To correct for non-uniformities in plate height, we measure the levitation gap on all four corners and obtain the average gap *H*. The results are shown in Fig. 2a, from which a clear reduction of the magnetic force with increasing gap *H* is observed. We also observe that the measurement errors vary for different data points, which is caused by the manual placement of the polymer blocks. Since we cannot easily reduce the gravitational force on the plate below *mg*, where *m* is the mass of the graphite plate without polymer blocks, the levitation gap cannot be raised above $H_0$, the equilibrium gap for which $F_m(H_0)=m g$.

To determine the full $F_m(H)$ curves, also for $H>H_0$, we perform analytic and Finite Element Method (FEM) calculations. The magnetic force of the four magnets on the diamagnetic plate can be analytically calculated using:

1 $$\begin{aligned} \textbf{F}_{\textbf{B}}&={\nabla } \int _\mathcal {V} \textbf{M} \cdot \textbf{B} \textrm{d}\mathcal {V} \nonumber \\&=\frac{\mu _0}{2} \int _\mathcal {V} {\nabla }(\chi _x H_x^2 + \chi _y H_y^2 +\chi _z H_z^2)\textrm{d}\mathcal {V}, \end{aligned}$$

where $\mathcal {V}$ is the volume of the plate, $H_{x,y,z}$ are the components of the magnetic field vector (*x* represents the vertical direction), $\textbf{M}$ is the magnetization vector and $\textbf{B}$ the magnetic flux density vector. In this analysis it is assumed that the plate does not significantly affect the magnetic field, since its relative magnetic permeability is close to 1. To calculate the magnetic force acting on the plate, we model the magnetic field of the four permanent magnets analytically using the charge model [28] and numerically using COMSOL Multiphysics (see details of modelling in Supporting Information S1 and S2). In Fig. 2a the calculated magnetic force $F_{\textrm{m}}$ is plotted as a function of the levitation gap *H* for both methods. It can be seen that the COMSOL simulations correspond well with the experimental data, while a small discrepancy is observed for the analytical model, especially for small values of *H*. This discrepancy is attributed to the fact that the edges of the cube magnets are not sharp, but slightly rounded, an effect that is included in the FEM simulation but not in the analytical model.

In the linear regime, the magnetic stiffness around the equilibrium position $H_0$ is obtained from the slope of the graph in Fig. 2a, $k_L=\frac{\textrm{d}F_z}{\textrm{d}H}={0.6625}$ N/m. Knowing the mass of the plate $m= {5.88 \times 10^{-5}}$ kg, the resonance frequency of the vertical rigid body mode of the plate is found to be $f_{\textrm{res}}=\frac{1}{2\pi }\sqrt{\frac{k_{\textrm{L}}}{m}}={16.89}$ Hz, which matches closely the measured value of $f_{\textrm{res}}={17.0}$ Hz (Fig. 1c).

However, for large amplitude motion, nonlinear terms in the magnetic force-displacement curve need to be taken into account. We describe the motion of the plate in terms of its displacement $x=H_0-H$ with respect to the equilibrium position, for the total restoring force given by $F_{\textrm{r}}=F_{\textrm{m}}-mg$. Therefore, $F_{\textrm{r}}(0)=0$, indicating that the gravitational force is canceled out in our following modeling and will not impact the resonator’s natural frequency and nonlinear dynamics. Figure 2b shows the potential energy obtained by $\int {F_{\textrm{r}}\textrm{d}x}$ using the simulated magnetic force (red circles in Fig. 2a). It is observed from Fig. 2b that interestingly, the potential well is not symmetric around the axis $x=0$, in contrast with many non-levitating mechanical systems that derive their nonlinear stiffness from nonlinear geometric effects. As a consequence, the center of motion (middle between maximum and minimum displacement) will be amplitude dependent and will not coincide with $H_0$ for large amplitude motion. For the maximal free vibration amplitude that the plate can sustain before colliding with the magnet, it displays a large asymmetry in its maximal displacement (zero kinetic energy) positions $x_\textrm{max}={1.18}$ mm and $x_\textrm{min}={-2.5}$ mm, as shown in Fig. 2b.

**Fig. 3:**
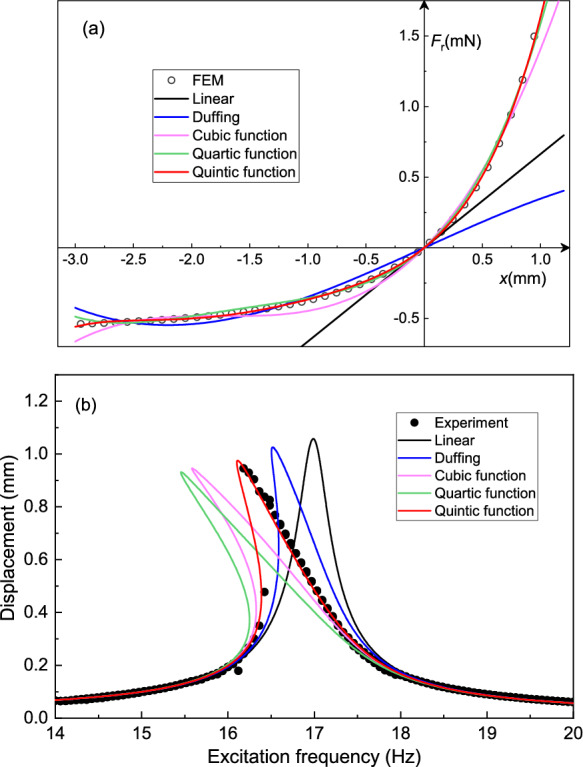
**a** Restoring force $F_{\textrm{r}}=F_{\textrm{m}}-mg$ of the levitating plate as a function of its displacement *x* when the plate is in free vibration. The hollow circles stand for the data obtained from FEM simulations and the lines are fits by polynomials from linear to quintic degree. **b** Frequency response curves of the plate with a driving voltage $V_{\textrm{ac}}={1.5}\,$V obtained by experiments (dots) and nonlinear dynamic simulations based on the polynomial stiffness functions from Fig. 3a (solid lines)

The restoring force $F_{\textrm{r}}$ of the plate as a function of its displacement *x* between ${-3.0}$ mm $<x<{1.2}$ mm, as simulated by FEM, is plotted in Fig. 3a. A linear fit of the data at $x=0$ is shown as a black solid line. It is interesting to note from Fig. 3a that, unlike conventional mechanical spring structures like double-clamped beams that have symmetric force-displacement curves for reflection around $x=0$ and other magnetic levitation systems [21, 23], the force-displacement curve of the levitating plate is asymmetric. Whereas asymmetries induced by external forces in nonlinear resonators [29, 30] have recently received interest from the community for affecting nonlinear parameters and inducing nonlinear phenomena like frequency combs, this asymmetry is intrinsic in the diamagnetically levitating plates, due to the strongly nonlinear magnetic field distributions above the permanent magnets in the vertical direction. As a consequence of the asymmetry, the force-displacement curve in Fig. 3a shows spring-hardening when $x>0$ and spring-softening when $x<0$, and this leads to an overall softening effect as seen in Fig. 1b, in contrast to other magnetic levitation systems reported in [21–23].

The asymmetry also implies that the magnetic force may not be fully described by a third order polynomial with Duffing-type nonlinear stiffness. To illustrate this, and determine the minimal degree of polynomial needed to capture the magnetic nonlinearity, we fit the FEM data around $x=0$ with polynomials from first to fifth degree in Fig. 3a, and list the fit parameters in Table 1. The functional form of the polynomials is $F_r=k_{\textrm{m1}}x+k_{\textrm{m2}}x^2+k_{\textrm{m3}}x^3+k_{\textrm{m4}}x^4+k_{\textrm{m5}}x^5$. We conclude from the fits that only the quintic, fifth degree polynomial fits well to the FEM data and thus we will use this function to construct the plate’s equation of motion and analyze its nonlinear dynamics.

**Table 1:** Fit parameters of the polynomial restoring force obtained from Fig. 3a

Function	$k_{\textrm{m1}}$ (N/m)	$k_{\textrm{m2}}$ (N/m$^{2}$)	$k_{\textrm{m3}}$ (N/m$^{3}$)	$k_{\textrm{m4}}$ (N/m$^{4}$)	$k_{\textrm{m5}}$ (N/m$^{5}$)
Linear	0.6625	–	–	–	–
Duffing	0.3732	–	-2.571 $\times 10^{4}$	–	–
Cubic	0.8110	479.4	9.381 $\times 10^{4}$	–	–
Quartic	0.7338	564.0	2.315 $\times 10^{5}$	3.549 $\times 10^{7}$	–
Quintic	0.6625	530	3.25 $\times 10^{5}$	1.114 $\times 10^{8}$	1.474 $\times 10^{10}$

### Dynamic modelling

After having determined the restoring force $F_{\textrm{r}}(x)$, the equation of motion of the plate under base excitation can be written as:

2 $$\begin{aligned} m\ddot{x}+c_{\textrm{e}}\dot{x}+F_{\textrm{r}}(x-y)=0. \end{aligned}$$

where $c_{\textrm{e}}$ is the linear damping coefficient due to eddy currents [7, 31], and $c_{\textrm{e}}=\frac{\sqrt{mk_{\textrm{m1}}}}{Q_{\textrm{L}}}={1.3 \times 10^{-4}}$ N s/m in this study; $y={d}\cos (\omega t)$ is the motion of the base as driven by the shaker. $C_\textrm{V}=1.58\times 10^{-2}\,$ mm/V is the conversion factor between the input voltage and base motion, such that the motion amplitude is given by ${d}=C_\textrm{V}V_{\textrm{ac}}$ (see also S3 for more details). For the quintic polynomial stiffness function, the nonlinear equation of motion is:

3 $$\begin{aligned}{} & {} m\ddot{x}+c_{\textrm{e}}\dot{x}+k_{\textrm{m1}}(x-y)+k_{\textrm{m2}}(x-y)^2 \nonumber \\{} & {} \quad +k_{\textrm{m3}}(x-y)^3+k_{\textrm{m4}}(x-y)^4+k_{\textrm{m5}}(x-y)^5=0. \nonumber \\ \end{aligned}$$

Next, we nondimensionalize the system using the natural levitation gap $H_0$ and the natural period *T*. In terms of the nondimensional variables $\hat{x}=\frac{x}{H_0}$ and $\hat{t}=\frac{t}{T}$, the nondimensional equation of motion becomes:

4 $$\begin{aligned}{} & {} \ddot{\hat{x}}+2\zeta _{\textrm{e}}\dot{\hat{x}}+\hat{x}-f_1\cos (\Omega \hat{t}) \nonumber \\{} & {} \quad +\alpha \left( \hat{x}-f_1\cos (\Omega \hat{t})\right) ^2+\beta (\hat{x}-f_1\cos (\Omega \hat{t}))^3 \nonumber \\{} & {} \quad +\alpha _2\left( \hat{x}-f_1\cos (\Omega \hat{t})\right) ^4+\beta _2(\hat{x}-f_1\cos (\Omega \hat{t}))^5=0, \nonumber \\ \end{aligned}$$

where $\alpha =\frac{k_{\textrm{m2}}H_0}{k_{\textrm{m1}}}$, $\beta =\frac{k_{\textrm{m3}}H_0^2}{k_{\textrm{m1}}}$, $\alpha _2=\frac{k_{\textrm{m4}}H_0^3}{k_{\textrm{m1}}}$, $\beta _2=\frac{k_{\textrm{m5}}H_0^4}{k_{\textrm{m1}}}$, $\zeta _{\textrm{e}}=\frac{c_{\textrm{e}}}{2\sqrt{k_{\textrm{m1}}m}}$, $f_1=\frac{{d}}{H_0}$ and $\Omega =\frac{\omega }{\omega _{\textrm{res}}}$. The linear resonance frequency of a resonator depends on its mass *m* and stiffness $k_{\textrm{m1}}$, given by $\omega _{\textrm{res}}=\sqrt{\frac{k_{\textrm{m1}}}{m}}$. In a system with only two stiffness terms $k_{\textrm{m1}}$ and $k_{\textrm{m2}}$, the frequency response curve will always bend to the left due to the softening effect of $k_{\textrm{m2}}\hat{x}^2$ no matter the sign of $k_{\textrm{m2}}$ [32]. In contrast, if the stiffness terms are $k_{\textrm{m1}}$ and $k_{\textrm{m3}}$, the nonlinear behavior will depend on the sign of $k_{\textrm{m3}}$: $k_{\textrm{m3}}>0$ results in a hardening effect, while $k_{\textrm{m3}}<0$ leads to a softening effect. When the stiffness involves more terms than the three cases mentioned above as in this study, the nonlinear behavior becomes more complex, resulting from the combined effect of all those terms.

**Fig. 4:**
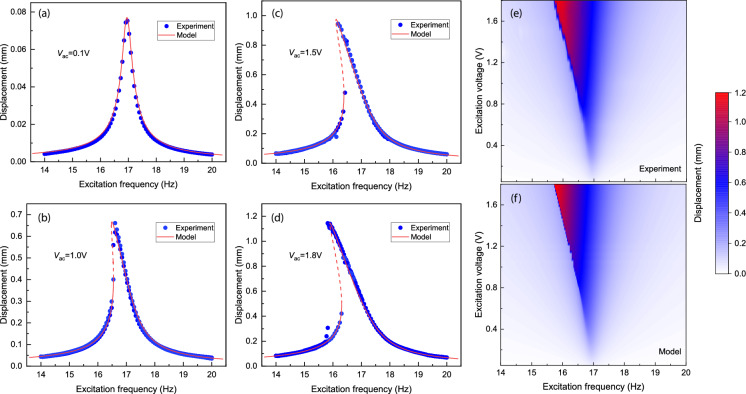
Comparison between the measured and modelled nonlinear frequency response of the levitating plate. **a**–**d** Experimental and modelled frequency response curves at four different driving voltages. The dots represent the experimental data and the lines represent the modelled data with stable (solid line) and unstable (dashed) solutions. Experimental (**e**) and modelled (**f**) frequency response curves with a wide range of driving voltages from 0.1 to 1.8 V, where the color represents the vibration amplitude

Using the stiffness parameters in Table 1, we solve equation Eq. (4) using a pseudo arc-length continuation technique [33] and obtain the amplitude-frequency curves for the 5 polynomial stiffness functions determined from Fig. 3a and plot them in Fig. 3b, comparing it to the experimental data (Fig. 1c) for a driving voltage $V_{\textrm{ac}}={1.5}$ V. We thus confirm that the quintic polynomial stiffness function corresponds well to the experimental response curve at this driving force, in contrast to lower order polynomial stiffness terms. We note that, even though the quartic function can capture the stiffness reasonably well (see Fig. 3a), it deviates substantially from the experimental frequency response curve at amplitude greater than 0.2 mm (see Fig. 3b).

In Figs. 4a–d the experimental data for different driving voltages are compared to simulations based on the equation of motion (4) and the quintic polynomial nonlinear magnetic force. It is noted from Fig.  4d that the plate motion nearly spans the full levitation gap of $H_0=$ 1.18mm, which demonstrates that our model captures the motion over this range with good accuracy. We also observe a slight discrepancy between the modeled and measured frequency response curves at frequencies near the bifurcation points. One possible reason for this is our assumption of a constant driving force in the model, whereas the experimental driving force is slightly frequency-dependent, as seen in Figure S5. Fig. 4e shows the experimental frequency response curves for all the curves shown in Fig. 1c which correspond well to the modelled curves in Fig. 4f over a large range of displacement. This correspondence provides confidence that nonlinear dynamics might also prove to be a useful tool for determining the nonlinear stiffness in levitating systems where no analytical models for the trap potential are available.

### Gas-induced nonlinear damping

**Fig. 5:**
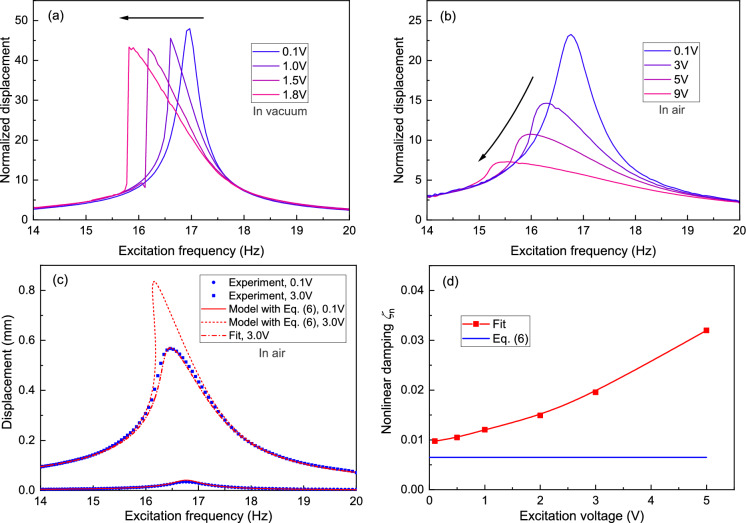
Air-induced nonlinear damping. **a** Normalized frequency response curves with different excitation voltages measured in vacuum at a pressure below ${5 \times 10^{-4}}$mbar where air damping is insignificant [7, 31]. **b** Normalized frequency response curves with different excitation voltages measured in air. **c** Frequency response curves with two excitation voltages measured in air and their corresponding modeled curves. The solid and dashed lines are modeled using Eqs. (5) and (6); the dash-dot line is modeled using Eq. (5) and taking $c_{\textrm{n}}$ as a fitting parameter. **d** Nonlinear damping ratio $\zeta _{\textrm{n}}$ as a function of driving voltages. The red line represents the damping ratio used in fitting the curves shown in Fig. S5. The blue line corresponds to the damping ratio calculated from Eq. (6)

After characterizing the nonlinear stiffness, we next study the nonlinearity of the damping in the levitating plate. It is known that eddy current forces dominate the damping mechanism in vacuum [7, 31] and determine the $c_{\textrm{e}}$ in Eq. 2. The fact that we obtained close agreement between experiment and model in Fig. 4 while using only a single quality factor $Q_{\textrm{L}}=48$, indicates that the eddy current damping force is quite linear, and proportional to the plate velocity. This can also be seen from Fig. 5a, where the normalized amplitude of motion $x_\textrm{max}/{d}$ is almost independent of the driving amplitude *d*, a signature that nonlinear terms in the eddy current damping force are small.

Since operating in air is favorable for many types of sensors, we next measure the levitating plate’s frequency response in open air to study the influence of air damping on nonlinear dynamics. The experimental procedure is similar to Fig. 1a, except that the experiments are conducted at atmospheric pressure at room temperature without the vacuum chamber. In Fig. 5b, we show normalized frequency response curves for four excitation forces. In contrast to the results measured in vacuum (Fig. 5a), a clear reduction in the normalized amplitude is observed when driving at higher voltages. This reduction is a clear signature of nonlinear damping, which originates from air damping [34]. Because the air gap between the levitating plate and the magnets is relatively small ($H_0={1.189}$ mm), it is likely that the major source of nonlinear damping is from the squeeze-film effect  [35], that is proportional to $\dot{x}/H^3$, and leads to the following equation of motion:

5 $$\begin{aligned} m\ddot{x}+c_{\textrm{e}}\dot{x}+\frac{c_{\textrm{n}}}{\left( 1-\frac{x-y}{H_0}\right) ^{3}}\dot{x}+F_{\textrm{r}}(x-y)=0, \end{aligned}$$

where $c_{\textrm{n}}$ is the nonlinear squeeze-film damping coefficient. Using Reynold’s equation, the nonlinear damping coefficient of a square plate can be written as [36]:

6 $$\begin{aligned} c_{\textrm{air}}=\frac{0.42\mu (L+\Delta L)^4}{H_0^3}, \end{aligned}$$

where $\mu ={1.825 \times 10^{-5}}$ kg/(m s) is the viscosity of air under atmospheric conditions, $L={10}$ mm is the side length of the square plate and $\Delta L=1.3H_0$ is the effective elongation of the plate taking into account the border effects.

Solving Eq. (5) using the damping coefficient in (6), the modeled frequency response matches quite well with the measurement when the driving force is small, as shown in the red solid line in Fig. 5c. When the driving force is small and the plate is in linear regime, the theoretical *Q* factor is $Q=\frac{\sqrt{k_\textrm{1}m}}{c_{\textrm{e}}+c_{\textrm{air}}}=29.6$, which is close to the measured linear $Q=24.8$. However, at high diving forces, the modeled results using the damping coefficient in Eq. (6) deviate from the experimental data significantly (see the dashed line in Fig. 5c). Therefore, to understand the variations of the squeeze-film damping coefficient as a function of drive amplitude, we take the nonlinear damping coefficient $c_{\textrm{n}}$ as a fit parameter (see the dash-dot line in Fig. 5c). We do the fitting for different drive levels and obtain the fitted nonlinear damping $\zeta _{\textrm{n}}=\frac{c_{\textrm{n}}}{2\sqrt{k_{\textrm{m1}}m}}$ as a function of excitation voltage, as shown in Fig. 5d (the fitting is shown in Fig. S7). We note from Fig. S7 that with air damping present, the dynamic range is limited to 0.57 mm. Beyond this range, the model fails to fully capture the frequency response curve of the resonator. We can also see from Fig. 5d that with increasing driving force, the normalized damping ratio increases from 0.01 to 0.03, deviating from the theoretical value more with increasing amplitude. The discrepancy is mainly due to the fact that the squeeze model in Eqs. (5) and (6) is only valid for relatively small motion amplitudes [34], while the motion of our levitating resonator is at such a high amplitude that can cover the whole air gap. Another influencing factor is that the surface of the magnets is not perfectly flat with trenches between the magnets (see Fig. 1b), which will make a difference in the boundary condition of the squeeze-film model. Therefore, to capture the whole nonlinear damping of the levitating plate in large amplitude vibrations in open air, more sophisticated nonlinear damping models or finite element methods shall be employed.

## Conclusions

In conclusion, we have explored the nonlinear dynamics of a milli-scale diamagnetically levitating graphite plate. Using gravitational force, we characterize the nonlinearity of the magnetic force field in which the plate is trapped. With dynamic measurements, we observe that the intrinsic nonlinearity of the repulsive magnetic force in the diamagnetic levitation system causes a spring-softening effect that leads to lower peak frequency for increased driving, differentiating it from other magnetic levitation systems. Due to asymmetries in the magnetic-gravitational potential, it is found that a quintic polynomial is needed to describe the force-displacement function with sufficient accuracy. Thus good agreement between experimental and simulated nonlinear dynamic frequency response is obtained in vacuum. Finally, we compare the normalized frequency response of the plate in air at atmospheric conditions, concluding that the eddy current damping is nearly linear and the squeeze film effect leads to strong nonlinear damping. This study of the nonlinear dynamics of levitating systems provides insight into the effects of the nonlinear stiffness of a magnetic trap and air damping forces on the nonlinear motion of levitating objects in the presence of base excitation. Moreover, it demonstrates an approach for analyzing the nonlinear dynamics of other levitating systems that will likely become of increasing relevance considering the growing interest in the field of levitodynamics and levitated optomechanics [1, 10, 37, 38].

### Supplementary Information

Below is the link to the electronic supplementary material.

Supplementary file 1 (pdf 1868 KB)

## Data Availability

The data that support the findings of this study are available from the corresponding author upon reasonable request.

## References

[1] Gonzalez-Ballestero, C., Aspelmeyer, M., Novotny, L., Quidant, R., Romero-Isart, O.: Levitodynamics: levitation and control of microscopic objects in vacuum. Science **374**, eabg3027 (2021) 34618558 10.1126/science.abg3027

[2] Delić, U., Reisenbauer, M., Dare, K., Grass, D., Vuletić, V., Kiesel, N., Aspelmeyer, M.: Cooling of a levitated nanoparticle to the motional quantum ground state. Science **367**, 892 (2020) 32001522 10.1126/science.aba3993

[3] Magrini, L., Rosenzweig, P., Bach, C., Deutschmann-Olek, A., Hofer, S.G., Hong, S., Kiesel, N., Kugi, A., Aspelmeyer, M.: Real-time optimal quantum control of mechanical motion at room temperature. Nature **595**, 373 (2021) 34262213 10.1038/s41586-021-03602-3

[4] Millen, J., Deesuwan, T., Barker, P., Anders, J.: Nanoscale temperature measurements using non-equilibrium Brownian dynamics of a levitated nanosphere. Nat. Nanotechnol. **9**, 425 (2014) 24793558 10.1038/nnano.2014.82

[5] Lewandowski, C.W., Knowles, T.D., Etienne, Z.B., D’Urso, B.: High-sensitivity accelerometry with a feedback-cooled magnetically levitated microsphere. Phys. Rev. Appl. **15**, 014050 (2021) 10.1103/PhysRevApplied.15.014050

[6] Ranjit, G., Cunningham, M., Casey, K., Geraci, A.A.: Zeptonewton force sensing with nanospheres in an optical lattice. Phys. Rev. A **93**, 053801 (2016) 10.1103/PhysRevA.93.053801

[7] Chen, X., Keşkekler, A., Alijani, F., Steeneken, P.G.: Rigid body dynamics of diamagnetically levitating graphite resonators. Appl. Phys. Lett. **116**, 243505 (2020) 10.1063/5.0009604

[8] Xiong, F., Yin, P., Wu, T., Xie, H., Li, R., Leng, Y., Li, Y., Duan, C., Kong, X., Huang, P., et al.: Lens-free optical detection of thermal motion of a submillimeter sphere diamagnetically levitated in high vacuum. Phys. Rev. Appl. **16**, L011003 (2021) 10.1103/PhysRevApplied.16.L011003

[9] De Pasquale, G., Iamoni, S., Somà, A.: 3d numerical modeling and experimental validation of diamagnetic levitating suspension in the static field. Int. J. Mech. Sci. **68**, 56 (2013) 10.1016/j.ijmecsci.2012.12.018

[10] Tian, S., Jadeja, K., Kim, D., Hodges, A., Hermosa, G., Cusicanqui, C., Lecamwasam, R., Downes, J., Twamley, J.: Feedback cooling of an insulating high-q diamagnetically levitated plate. Appl. Phys. Lett. **124**, 124002 (2024) 10.1063/5.0189219

[11] Brandt, E.: Levitation in physics. Science **243**, 349 (1989) 17787252 10.1126/science.243.4889.349

[12] Jain, V., Gieseler, J., Moritz, C., Dellago, C., Quidant, R., Novotny, L.: Direct measurement of photon recoil from a levitated nanoparticle. Phys. Rev. Lett. **116**, 243601 (2016) 27367388 10.1103/PhysRevLett.116.243601

[13] Bullier, N., Pontin, A., Barker, P.: Characterisation of a charged particle levitated nano-oscillator. J. Phys. D Appl. Phys. **53**, 175302 (2020) 10.1088/1361-6463/ab71a7

[14] Wang, Q., Ren, X., Jiao, S., Lei, X., Zhang, S., Liu, H., Luo, P., Tu, L.: A diamagnetic levitation based inertial sensor for geophysical application. Sens. Actuators Phys. **312**, 112122 (2020) 10.1016/j.sna.2020.112122

[15] Timberlake, C., Gasbarri, G., Vinante, A., Setter, A., Ulbricht, H.: Acceleration sensing with magnetically levitated oscillators above a superconductor. Appl. Phys. Lett. **115**, 224101 (2019) 10.1063/1.5129145

[16] Middlemiss, R., Samarelli, A., Paul, D., Hough, J., Rowan, S., Hammond, G.: Measurement of the earth tides with a mems gravimeter. Nature **531**, 614 (2016) 27029276 10.1038/nature17397

[17] Schmöle, J., Dragosits, M., Hepach, H., Aspelmeyer, M.: A micromechanical proof-of-principle experiment for measuring the gravitational force of milligram masses. Class. Quantum Gravity **33**, 125031 (2016) 10.1088/0264-9381/33/12/125031

[18] Chen, X., Kothari, N., Keşkekler, A., Steeneken, P.G., Alijani, F.: Diamagnetically levitating resonant weighing scale. Sens. Actuators Phys. **330**, 112842 (2021) 10.1016/j.sna.2021.112842

[19] Leng, Y., Li, R., Kong, X., Xie, H., Zheng, D., Yin, P., Xiong, F., Wu, T., Duan, C.-K., Du, Y., et al.: Mechanical dissipation below 1 HZ with a cryogenic diamagnetic levitated micro-oscillator. Phys. Rev. Appl. **15**, 024061 (2021) 10.1103/PhysRevApplied.15.024061

[20] Gieseler, J., Novotny, L., Quidant, R.: Thermal nonlinearities in a nanomechanical oscillator. Nat. Phys. **9**, 806 (2013) 10.1038/nphys2798

[21] Mann, B., Sims, N.: Energy harvesting from the nonlinear oscillations of magnetic levitation. J. Sound Vib. **319**, 515 (2009) 10.1016/j.jsv.2008.06.011

[22] Liu, L., Yuan, F.: Nonlinear vibration energy harvester using diamagnetic levitation. Appl. Phys. Lett. **98**, 203507 (2011) 10.1063/1.3583675

[23] Liu, L., Yuan, F.: Diamagnetic levitation for nonlinear vibration energy harvesting: theoretical modeling and analysis. J. Sound Vib. **332**, 455 (2013) 10.1016/j.jsv.2012.08.004

[24] Guccione, G., Hosseini, M., Adlong, S., Johnsson, M., Hope, J., Buchler, B., Lam, P.K.: Scattering-free optical levitation of a cavity mirror. Phys. Rev. Lett. **111**, 183001 (2013) 24237512 10.1103/PhysRevLett.111.183001

[25] Vikrant, K., Jayanth, G.: Diamagnetically levitated nanopositioners with large-range and multiple degrees of freedom. Nat. Commun. **13**, 3334 (2022) 35680887 10.1038/s41467-022-31046-4 PMC9184538

[26] Ahn, J., Xu, Z., Bang, J., Ju, P., Gao, X., Li, T.: Ultrasensitive torque detection with an optically levitated nanorotor. Nat. Nanotechnol. **15**, 89 (2020) 31932762 10.1038/s41565-019-0605-9

[27] Xu, Y., Cui, Q., Kan, R., Bleuler, H., Zhou, J.: Realization of a diamagnetically levitating rotor driven by electrostatic field. IEEE/ASME Trans. Mechatron. **22**, 2387 (2017) 10.1109/TMECH.2017.2718102

[28] Furlani, E.P.: Permanent Magnet and Electromechanical Devices: Materials, Analysis, and Applications. Academic Press, Cambridge (2001)

[29] Ochs, J.S., Rastelli, G., Seitner, M., Dykman, M.I., Weig, E.M.: Resonant nonlinear response of a nanomechanical system with broken symmetry. Phys. Rev. B **104**, 155434 (2021) 10.1103/PhysRevB.104.155434

[30] Keskekler, A., Arjmandi-Tash, H., Steeneken, P.G., Alijani, F.: Symmetry-breaking-induced frequency combs in graphene resonators. Nano Lett. **22**, 6048 (2022) 35904442 10.1021/acs.nanolett.2c00360 PMC9373031

[31] Chen, X., Ammu, S.K., Masania, K., Steeneken, P.G., Alijani, F.: Diamagnetic composites for high-q levitating resonators. Adv. Sci. **9**, 2203619 (2022) 10.1002/advs.202203619 PMC9661851 36180390

[32] Nayfeh, A.H., Mook, D.T.: Nonlinear Oscillations. Wiley, New York (2008)

[33] Doedel, E.J., Champneys, A.R., Fairgrieve, T.F., Kuznetsov, Y.A., Sandstede, B., Wang, X., et al.: Auto97, Continuation and bifurcation software for ordinary differential equations ( 1998)

[34] Bao, M., Yang, H.: Squeeze film air damping in mems. Sens. Actuators Phys. **136**, 3 (2007) 10.1016/j.sna.2007.01.008

[35] Sadd, M.H., Stiffler, A.K.: Squeeze film dampers: amplitude effects at low squeeze numbers. J. Eng. Ind. **97**, 1366 (1975). 10.1115/1.3438789 10.1115/1.3438789

[36] Veijola, T., Pursula, A., Råback, P.: Extending the validity of squeezed-film damper models with elongations of surface dimensions. J. Micromech. Microeng. **15**, 1624 (2005) 10.1088/0960-1317/15/9/003

[37] Winstone, G., Bhattacharya, M., Geraci, A.A., Li, T., Pauzauskie, P.J., Vamivakas, N.: Levitated optomechanics: a tutorial and perspective. arXiv:2307.11858 (2023)

[38] Dania, L., Bykov, D.S., Goschin, F., Teller, M., Northup, T.E.: Ultra-high quality factor of a levitated nanomechanical oscillato. arXiv:2304.02408 (2023) 10.1103/PhysRevLett.132.133602 38613288

