# Supplementary material for: Nonlinear dynamics of diamagnetically levitating resonators
Source: Nonlinear Dyn. 2024 Jul 20;112(21):18807–16. doi: 10.1007/s11071-024-10018-x (PMC11362389; doi:10.1007/s11071-024-10018-x)
Supplement: Supplementary file 1 — (pdf 1868 KB) [file 11071_2024_10018_MOESM1_ESM.pdf]

Supporting Information:
Nonlinear dynamics of diamagnetically levitating resonators

Xianfeng Chen,[1,2] Tjebbe de Lint,[1] Farbod Alijani,[1] and Peter G. Steeneken[1,3]

[1]*Department of Precision and Microsystems Engineering, Delft University of Technology, Mekelweg 2, 2628 CD, Delft, The Netherlands*

[2]*A*STAR Quantum Innovation Centre(Q.InC), Institute for Materials Research and Engineering(IMRE), Agency for Science, Technology and Research(A*STAR), 2 Fusionopolis Way, 08-03 Innovis 138634, Singapore*

[3]*Kavli Institute of Nanoscience, Delft University of Technology, Lorentzweg 1, 2628 CJ, Delft, The Netherlands.*

(Dated: July 16, 2024)

S1. MAGNETIC FIELD CALCULATION BY ANALYTICAL MODELING

In this section, we determine the magnetic field $\mathbf{B}$ outside a rectangular permanent magnet using the charge model following the derivation in reference [1]. We first introduce the charge model of determining the magnetic field of a unit magnet source, and then expand it to one rectangular permanent magnet.

A. The charge model

The charge model is a useful method to calculate the magnetic field distribution of permanent magnets. In this model, a magnet is taken as a distribution of equivalent 'magnetic charge', which is the source of magnetic field. For a current-free region and magnetostatic field, $\nabla \times \mathbf{H} = 0$ and $\nabla \cdot \mathbf{B} = 0$, where $\mathbf{H}, \mathbf{B}$ are the magnetic field strength and magnetic flux density due to a magnetic source, respectively. Then, the irrotational magnetic vector field $\mathbf{H}$ can be written as the gradient of the magnetic scalar potential ϕ_m:

$$\mathbf{H} = -\nabla \phi_\mathrm{m}. \tag{1}$$

Because $\mathbf{B} = \mu_0(\mathbf{H} + \mathbf{M})$, where μ_0 is the vacuum permeability and $\mathbf{M}$ is the magnetization, combining Eq. (1) and $\nabla \cdot \mathbf{B} = 0$ results in:

$$\nabla^2 \phi_\mathrm{m} = \nabla \cdot \mathbf{M}. \tag{2}$$

For a free space without boundary conditions, we can solve Eq. (2) using Green's function $G(\mathbf{x}, \mathbf{x_0})$ for ∇^2 and obtain a particular solution:

$$\phi_\mathrm{m}(\mathbf{x}) = \int G(\mathbf{x}, \mathbf{x_0}) \nabla' \cdot \mathbf{M}(\mathbf{x_0}) \mathrm{d}v_0 \tag{3}$$

$$= -\frac{1}{4\pi} \int \frac{\nabla' \cdot \mathbf{M}(\mathbf{x_0})}{|\mathbf{x} - \mathbf{x_0}|} \mathrm{d}v_0, \tag{4}$$

where $\mathbf{x}$ is the observation point, $\mathbf{x_0}$ is the source point, ∇' operates on the primed coordinates, and the integration is over the volume for which the magnetization exists. Assuming $\mathbf{M}$ is confined in a volume V with boundary surface S and falls abruptly to zero outside of this volume, Eq. (4) can be written as:

$$\phi_\mathrm{m}(\mathbf{x}) = -\frac{1}{4\pi} \int_V \frac{\nabla' \cdot \mathbf{M}(\mathbf{x_0})}{|\mathbf{x} - \mathbf{x_0}|} \mathrm{d}v_0 + \frac{1}{4\pi} \oint_S \frac{\mathbf{M}(\mathbf{x_0}) \times \hat{\mathbf{n}}}{|\mathbf{x} - \mathbf{x_0}|} \mathrm{d}s_0, \tag{5}$$

where $\hat{\mathbf{n}}$ is the outward unit normal to S. Therefore, the volume charge densities ρ_m and surface charge densities σ_m can be defined as:

$$\rho_\mathrm{m} = -\nabla \cdot \mathbf{M} \tag{6}$$

$$\sigma_\mathrm{m} = \mathbf{M} \cdot \hat{\mathbf{n}}. \tag{7}$$

For free space, $\mathbf{B} = \mu_0 \mathbf{H}$, and subtituting Eq. (5) into Eq. (1) obtains:

$$\mathbf{B}(\mathbf{x}) = \frac{\mu_0}{4\pi} \int_V \frac{\rho_\mathrm{m}(\mathbf{x_0})(\mathbf{x} - \mathbf{x_0})}{|\mathbf{x} - \mathbf{x_0}|^3} \mathrm{d}v_0 + \frac{\mu_0}{4\pi} \oint_S \frac{\sigma_\mathrm{m}(\mathbf{x_0})(\mathbf{x} - \mathbf{x_0})}{|\mathbf{x} - \mathbf{x_0}|^3} \mathrm{d}s_0, \tag{8}$$

B. Magnetic field of one cube permanent magnet

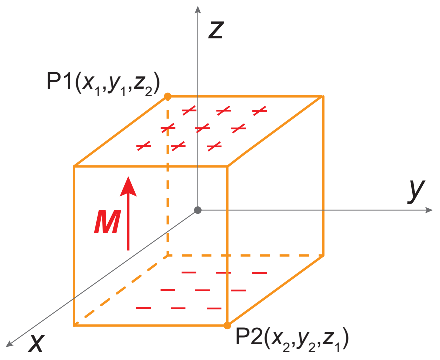

FIG. S1. Schematic of one permanent magnet with magnetization **M** and its corresponding coordinate system. The charge densities on the top and bottom surfaces of the magnet are represented with + and -, respectively.

For a permanent magnet as shown in Fig. S1, assuming that its magnetization is $\mathbf{M} = M_\mathrm{r}\hat{z}$ along z direction and its dimension can be denoted by two points $\mathrm{P1}(x_1, y_1, z_2)$ and $\mathrm{P2}(x_2, y_2, z_1)$, its magnetic field $\mathbf{B}$ can be calculated using the charge model. For permanent magnets, the volume charge density inside the magnets is zero $\rho_\mathrm{m} = -\nabla \cdot \mathbf{M} = 0$, while the surface charge densities on top and bottom surfaces are:

$$\sigma_\mathrm{m} = \begin{cases} M_\mathrm{r} & (z = z_2) \\ -M_\mathrm{r} & (z = z_1). \end{cases} \tag{9}$$

Based on Eq. (8), the magnetic field of the rectangular magnet is:

$$\mathbf{B}(x,y,z) = \frac{\mu_0 M_\mathrm{r}}{4\pi}\sum_{k=1}^{2}(-1)^k \times \int_{y_1}^{y_2}\int_{x_1}^{x_2}\frac{[(x-x_0)\hat{\mathbf{x}} + (y-y_0)\hat{\mathbf{y}} + (z-z_k)\hat{\mathbf{z}}]\mathrm{d}x_0\mathrm{d}y_0}{[(x-x_0)^2+(y-y_0)^2+(z-z_k)^2]^{3/2}}. \tag{10}$$

Therefore, by integrating Eq. (10) with respect to x_0 and y_0, we can obtain the x-component of the magnetic field:

$$B_x(x,y,z) = \frac{\mu_0 M_\mathrm{r}}{4\pi}\sum_{k=1}^{2}\sum_{m=1}^{2}(-1)^{k+m}\ln[\frac{(y-y_1)+[(x-x_m)^2+(y-y_1)^2+(z-z_k)^2]^{1/2}}{(y-y_2)+[(x-x_m)^2+(y-y_2)^2+(z-z_k)^2]^{1/2}}], \tag{11}$$

and the y-component of the magnetic field:

$$B_y(x,y,z) = \frac{\mu_0 M_\mathrm{r}}{4\pi}\sum_{k=1}^{2}\sum_{m=1}^{2}(-1)^{k+m}\ln[\frac{(x-x_1)+[(x-x_1)^2+(y-y_m)^2+(z-z_k)^2]^{1/2}}{(x-x_2)+[(x-x_2)^2+(y-y_m)^2+(z-z_k)^2]^{1/2}}]. \tag{12}$$

Similarly, the z-component of the magnetic field can be obtained:

$$B_z(x,y,z) = \frac{\mu_0 M_\mathrm{r}}{4\pi}\sum_{k=1}^{2}\sum_{n=1}^{2}\sum_{m=1}^{2}(-1)^{k+n+m} \tag{13}$$

$$\times \tan^{-1}[\frac{(x-x_n)(y-y_m)}{z-z_k}\frac{1}{[(x-x_n)^2+(y-y_m)^2+(z-z_k)^2]^{1/2}}]. \tag{14}$$

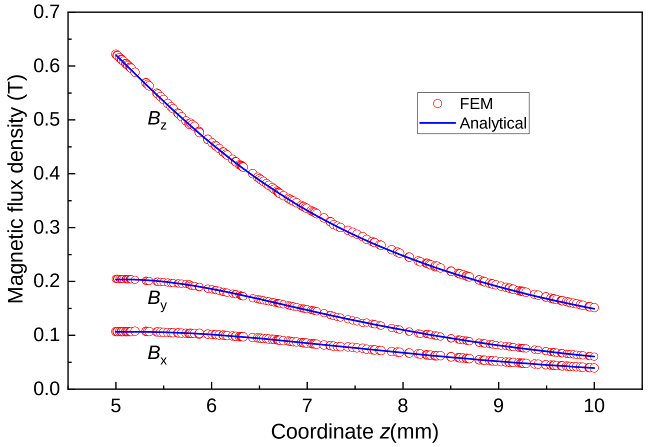

FIG. S2. Schematic of one magnet with magnetization **M** and its corresponding coordinate system.

Normally, the magnetic property of a permanent magnet is described by its remanent magnetic flux density B_{r}, and $B_{\mathrm{r}} = \mu_0 M_{\mathrm{r}}$. Therefore, with a known B_{r} and dimensions, the magnetic field outside a permanent magnet can be calculated using Eq. (11), Eq. (12) and Eq. (13). For example, for a magnet with $B_{\mathrm{r}} = 1.4\,\mathrm{T}$ and dimensions of $10 \times 10 \times 10\,\mathrm{mm}^3$ ($x_1 = -5, x_2 = 5, y_1 = -5, y_2 = 5, z_1 = -5, z_2 = 5$), its magnetic field B_x, B_y, B_z along a line ($x = 2\,\mathrm{mm}, y = 3\,\mathrm{mm}, 5\,\mathrm{mm} < z < 10\,\mathrm{mm}$) are calculated using Eq. (11-13) and shown in Fig. S2. For comparison, we also use a finite element method (COMSOL Multiphysics) to calculate the magnetic field of the same magnet and the results are also shown in Fig. S2, from which a good agreement between the two methods is observed.

C. Magnetic field of an array of magnets

For an array of multiple permanent magnets, the magnetic field in free space can also be determined using Eq. (11-13). For example, for a magnet array as shown in Fig. S3, it magnetic field can be calculated by:

$$\mathbf{B}(x, y, z) = \sum_{i=1}^{4} \mathbf{B}_i(x, y, z), \tag{15}$$

where $\mathbf{B}_i(x, y, z)$ is the magnetic filed of the $i-$th magnet.

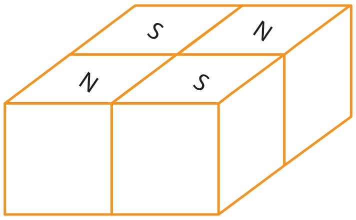

FIG. S3. An array of four permanent magnets with alternating magnetic poles, where 'N' represents the north pole and 'S' the south pole, respectively.

After the magnetic field is determined, the magnetic force applied on the graphite plate can be calculated with:

$$\begin{aligned}\mathbf{F_B} &= \nabla \int_{\mathcal{V}} \mathbf{M} \cdot \mathbf{B} \mathrm{d}\mathcal{V} \\ &= \frac{\mu_0}{2} \int_{\mathcal{V}} \nabla(\chi_x H_x^2 + \chi_y H_y^2 + \chi_z H_z^2) \mathrm{d}\mathcal{V},\end{aligned} \tag{16}$$

where $\mathcal{V}$ is the volume of the plate, $H_{x,y,z}$ are the components of the magnetic field vector, **M** is the magnetization vector and **B** the magnetic flux density vector. In this analysis it is assumed that the plate does not significantly affect

TABLE I. Material properties used for the simulations of the levitating pyrolytic graphite.

Property	Symbol	Value	Unit
Density	ρ	2070	kg/m^3
Susceptibility ⊥ [2]	χ_z	-450	$\times 10^{-6}$
Susceptibility ∥ [2]	$\chi_{x,y}$	-85	$\times 10^{-6}$

the magnetic field, since its relative magnetic permeability is close to 1. All the parameters used in our calculations for pyrolytic graphite plates are listed in Table I.

S2. MAGNETIC FIELD CALCULATION BY FEM MODELING

In order to compare with the analytical modeling and take into account the fillets on the edges of the magnets, we also use FEM method to calculate the magnetic force. The geometry model of the magnets and graphite plate is shown in Fig. S4. The magnets are with dimensions of $12 \times 12 \times 12\,\mathrm{mm}^3$ and with rounded edges with a fillet radius of 1 mm. The remanent magnetic flux density of the magnets is $B_\mathrm{r} = 1.4\,\mathrm{T}$. The material properties of the $10 \times 10 \times 0.28\,\mathrm{mm}^3$ graphite plate are listed in Table I. Using *COMSOL Multiphysics 5.6*, we can simulate the magnetic field and then calculate the magnetic force using Eq. (16).

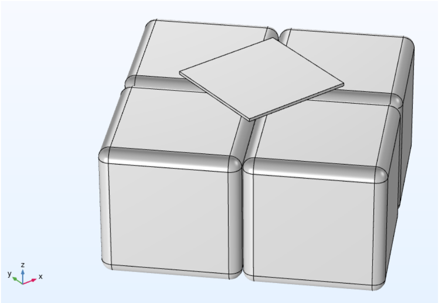

FIG. S4. Geometry model of one graphite plate levitating over four permanent magnets.

S3. CHARACTERIZATION OF SHAKER

To determine the relations between the output motion and driving voltage of the shaker, we shine the laser directly on the magnets and measure their displacement amplitude d with different driving voltages V_ac. The measurement results for 4 different driving frequencies are shown in Fig. S5. It can be seen that the displacement of the shaker is approximately linear with the driving voltage and $d = C_\mathrm{V} V_\mathrm{ac}$, where $C_\mathrm{V} = 0.0158\mathrm{mm/V}$. We note that the slope of the conversion factor C_V increases from 0.0152 to 0.0178 when the frequency increases from 14Hz to 20Hz, which is attributed to the shaker having a slightly frequency-dependent base amplitude.

S4. SOLVING PROCEDURES FOR NONLINEAR EQUATIONS OF MOTION

The equation of motion was solved using a pseudo arc-length continuation technique implemented in the numerical software AUTO, with detailed documentation available in the manual [3] and on GitHub [4]. Here, we only summarize the key solving procedures:

(1) Transform the equation of motion (Eq. (4) in the main text) into two first-order differential equations. Input these equations along with all normalized physical parameters into the parameter setting file of AUTO.

(2) Set the control file of AUTO. The important settings for our system are:

- **NDIM=1**: Indicates only one degree of freedom.
- **IPS=2**: Specifies the computation of periodic solutions.

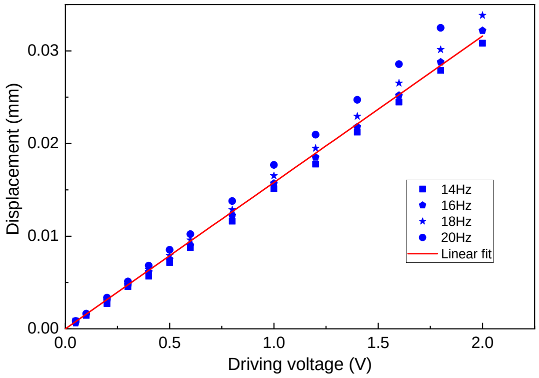

FIG. S5. Displacement amplitude of the shaker as a function of driving voltages for four driving frequencies.

- **DSMIN=0.000001** and **DSMAX=0.005**: Set the minimal and maximal pseudo-arclength step sizes for the first attempted step along any branch. This is important since our system has a bifurcation point.
- Initialize the sweeping frequency and define the sweeping frequency range.

(3) Retrieve the amplitude of the periodic solution at each step and plot the frequency response curve.

The stability analysis of the periodic solutions is done also via AUTO and by checking the Floquet multipliers associated with the periodic solution. More details on these can be found in our earlier work [5], as well as [6]

S4. MODELING NONLINEAR FREQUENCY RESPONSE IN AIR

Taking the nonlinear damping coefficient as a fit parameter, we can model the nonlinear frequency response curves using Eq. (5) in the main text, which match the experiment results well, as shown in Fig. S7. Note that to obtain a good fit, we need to adjust the liner resonance frequency for each fitting. The linear resonance frequencies used for the frequency response curve fitting are listed in Table II.

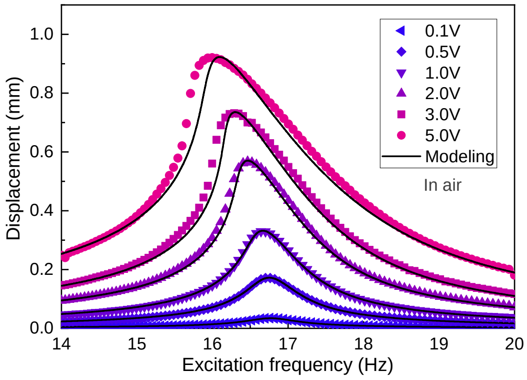

FIG. S6. Frequency response curves with different excitation voltages measured in air and their corresponding modeled curves using Eq. (5) in the main text.

TABLE II. Linear resonance frequencies used for the frequency response curve fitting

Driving voltage (V)	0.1	0.5	1	2	3	5
Resonance frequency (Hz)	16.78	16.80	16.82	16.86	16.89	16.92

[1] E. P. Furlani, *Permanent magnet and electromechanical devices: materials, analysis, and applications* (Academic press, 2001).

[2] M. Simon and A. Geim, Diamagnetic levitation: Flying frogs and floating magnets, Journal of applied physics **87**, 6200 (2000).

[3] E. J. Doedel, A. R. Champneys, T. F. Fairgrieve, Y. A. Kuznetsov, B. Sandstede, X. Wang, *et al.*, Auto97, Continuation and bifurcation software for ordinary differential equations (1998).

[4] https://github.com/auto-07p/auto-07p, .

[5] B. Sajadi, F. Alijani, H. Goosen, and F. van Keulen, Effect of pressure on nonlinear dynamics and instability of electrically actuated circular micro-plates, Nonlinear Dynamics **91**, 2157 (2018).

[6] A. H. Nayfeh and B. Balachandran, *Applied nonlinear dynamics: analytical, computational, and experimental methods* (John Wiley & Sons, 2008).
